# Diagnostic value of ^18^F-FDG PET/CT as first choice in the detection of recurrent colorectal cancer due to rising CEA

**DOI:** 10.1186/s40644-015-0048-y

**Published:** 2015-08-13

**Authors:** Michael Gade, Magdalena Kubik, Rune V. Fisker, Ole Thorlacius-Ussing, Lars J. Petersen

**Affiliations:** The Department of Nuclear Medicine, Clinical Cancer Research Center, Aalborg University Hospital, Hobrovej 18-22, DK-9000 Aalborg, Denmark; The Department of Radiology, Clinical Cancer Research Center, Aalborg University Hospital, Aalborg, Denmark; The Department of Gastrointestinal Surgery, Clinical Cancer Research Center, Aalborg University Hospital, Aalborg, Denmark; The Department of Clinical Medicine, Aalborg University, Aalborg, Denmark

**Keywords:** Colorectal cancer, ^18^F-FDG, PET/CT, CEA, Recurrence, Radical surgery

## Abstract

**Background:**

The diagnostic value of ^18^F-fluorodeoxyglucose positron emission tomography/computed tomography (^18^F-FDG PET/CT) as the first imaging approach in the evaluation of rising carcinoembryonic antigen (CEA) is not clear. The objective of this study was to investigate the value of ^18^F-FDG PET/CT in patients with colorectal cancer (CRC) and suspected recurrence based on rising CEA.

**Methods:**

A total of 73 patients with CRC were referred to PET/CT after radical surgery. Generally, all patients were scheduled to follow a CT-based post-surgical follow-up regimen. In the case of rising CEA, ^18^F-FDG PET/CT was performed in most patients with contrast-enhanced CT. The PET/CT images were independently reviewed by two readers. The presence or absence of recurrence was based on histology and/or standardized clinical follow-up.

**Results:**

Among 35 patients who had confirmed recurrence of CRC, PET/CT demonstrated recurrence with a sensitivity of 85.7 %, a specificity of 94.7 %, a positive predictive value of 93.8 %, and a negative predictive value of 87.8 %. The SUV_max_ ranged from 1.3 to 19.9. The mean time since the last postoperative imaging and PET/CT was 8 months (median 4 months). CEA values at referral ranged from 1.5 to 164.0 μg/L (median 5.6 μg/L). The diagnostic properties of PET/CT were analyzed in subgroups of patients with a single rising CEA sample (30 patients, 41 %), 31 patients (43 %) with two or more consecutive increases, and 12 patients (16 %) with persistently elevated values.

**Conclusions:**

^18^F-FDG PET/contrast-enhanced CT has high diagnostic accuracy in the diagnosis of recurrent CRC, even in patients in a conventional CT-based follow-up program.

## Background

Colorectal cancer (CRC) is the third most common cancer in the world. It is estimated that up to 40 % of patients will present with recurrence after surgical resection of the primary tumor, often within 2 years [1]. Imaging plays a key role in the postoperative assessment of recurrent disease. Most guidelines recommend computed tomography (CT) at regular intervals, e.g. thoracoabdominal CT at 12 and 36 months postoperatively as well as upon clinical suspicion.

The role of ^18^F-fluorodeoxyglucose positron emission tomography/computed tomography (^18^F-FDG PET/CT) in the follow-up of CRC patients is controversial. Recent systematic reviews suggest no indication for ^18^FDG-PET/CT except in cases of inconclusive CT [2, 3] or suspicion of distant metastasis [4].

The role of CEA as a biomarker for imaging in CRC is not clear. CEA is expressed in the majority of CRC cells [5], but elevated CEA may be due to a variety of benign and malignant conditions [6]. However, unexplained rising CEA after surgery is a strong indicator of CRC recurrence [7, 8]. A recent meta-analysis showed an excellent sensitivity of 94 % and acceptable specificity of 77 % of ^18^F-FDG PET/CT for recurrent CRC in patients with rising CEA [9].

US oncology guidelines recommend PET/CT in the presence of negative CT and serial CEA increases. The role of CT as a gate-keeper for PET/CT is not clear.

It has been our institutional practice for years to perform ^18^FDG PET/CT as the first modality for clinical suspicion of recurrent CRC based on rising or abnormal CEA, i.e. without a prior CECT. Here we report excellent diagnostic properties for ^18^FDG PET/CT, including in patients with a recent CECT as part of routine follow up. The details of CEA, i.e. the number of rises and the CEA values at referral, were analyzed and showed value of PET/CT among most patients.

## Methods

### Patients

We retrospectively identified consecutive patients referred for PET/CT on the suspicion of recurrent CRC. The patients had an ^18^F-FDG PET/CT scan performed between January 1, 2006 and May 28, 2013 at our department. The eligibility criteria were as follows: 1) Histologically confirmed diagnosis of CRC, 2) pathology-verified radical surgery, 3) suspicion of recurrent CRC due to at least one rising CEA or CEA above upper limit of normal. Patients referred for PET/CT due to negative or inconclusive results of prior imaging performed due to clinical suspicion (not routine scans as part of follow up programs) were excluded. If eligible patients had more than one PET/CT due to rising CEA, only the first procedure was included in the analysis. A few patients were treated with non-surgically, e.g. by radiofrequency ablation of synchronous or metachronous liver metastasis and considered curatively treated based on clinical and imaging follow-up.

### Postoperative surveillance program

Most patients (>85 %) followed a standardized post-operative follow-up program, which included CT 12 and 36 months postoperatively. A total of 18 of the patients participated in the COLOFOL study [10], of which 9 patients received standard CT follow up, and 9 patients had CT scheduled at 6, 12, 18, 24 and 36 months.

### CEA

The measurement of serial CEA levels was integrated in the post-surgical monitoring. The time intervals between CEA measurements varied among patients due to individual baseline CEA, CEA doubling time, or CEA velocity (increase in μg/L per time unit). Thus, no definite CEA cut-off value was required for referral for PET/CT. Patients were divided into quartiles based on CEA levels. The upper limit of normal for CEA was 3.0 μg/L for non-smokers (*n* = 65) and 10.0 μg/L for smokers (*n* = 8).

### Imaging procedure and evaluation

Whole-body images were obtained by PET/CT (Discovery VCT, GE Healthcare) in accordance with institutional procedures. The mean dose was 379 MBq of ^18^F-FDG. Blood glucose levels were less than 11 mmol/L in all patients. The PET/CT scan was acquired approximately 60 min after tracer injection. Low-dose CT was acquired in 9 (12 %) patients, and diagnostic CT with intravenous iodinated contrast was performed in 64 patients (88 %). Most of the latter patients received 100 ml of Iomeron® 400 mg iodine/mL (*n* = 52).

Due to inconsistent reporting of maximum standardized uptake values (SUV_max_) in clinical study reports, all images were independently reviewed by two radiologists with solid experience with PET/CT. Any discrepancy in diagnosis among the readers was solved by consensus. The readers were aware of the clinical history and laboratory results at referral but blinded to the original PET/CT report, clinical follow-up, and pathology reports. The classification of lesions as malignant or benign on PET/CT was based on combined parameters from both PET and CT. The readers did not report the diagnostic outcome for each modality separately. In a few cases, the PET/CT was classified as malignant based on CT findings only, e.g. small pulmonary nodules.

### Definition of recurrence

The presence or absence of recurrent CRC was determined based on histopathological examination or follow up. In the absence of positive pathology, we aimed at a clinical follow up of at least 24 months of observation with conventional imaging modalities showing progression in measurable lesions by RECIST criteria, or by an adequate response to therapeutic interventions to clarify recurrent CRC. The absence of disease was established if no malignancy could be detected with available information regarding additional imaging, serial physical examinations including laboratory quantities and regular endoscopy without abnormalities. The results were performed on a patient basis.

### Statistical analysis

Data were described by mean or median with total range and proportions. Statistical analysis was performed using Chi square test using GraphPad Prism version 6.05 (GraphPad Software, Inc., California, USA). Two-sided p-values <0.05 were considered statistically significant.

### Ethical approval and consent

This retrospective study did not require ethical approval or informed consent in accordance with national legislation. The study was approved by the Danish Data Protection Agency who provided waiver for informed consent for access to medical files and to publish the findings.

## Results

### Patients

The final study population consisted of 73 patients (Table 1). The histology showed adenocarcinoma in all patients. In addition to surgery, eight patients received additional curative intervention for synchronous metastasis (*n* = 6) or metachronous metastasis (*n* = 2). The mean time since curative surgery and the PET/CT scan was 24 months (range 2–84 months). The mean time between the measurement of CEA and the PET/CT scan was 34 days (range 8–111 days). Thirty-five patients (48 %) had a recurrence of CRC of which 25 were confirmed by pathology and 10 by clinical follow-up. Thirty patients were followed without criteria for recurrent CRC for at least 24 months. Six patients were observed for 14–19 months without indications of recurrent disease. Two patients died within 24 months of PET/CT due to non-CRC cancer.

**Table 1:** Patient demographics and baseline information

Number of patients	73
Males	41 (56 %)
Females	32 (44 %)
Mean age, years (range)	65 (44–86)
Adenocarcinoma localization	
Caecum	6 (8 %)
Caecum and ascending colon	1 (≈1 %)
Ascending colon	4 (5 %)
Transverse colon	5 (7 %)
Descending colon	1 (≈1 %)
Sigmoid colon	13 (18 %)
Rectum	43 (59 %)
Primary tumor, pathological stage	
T1	5 (7 %)
T2	11 (15 %)
T3	39 (53 %)
T4	16 (22 %)
TX	2 (3 %)
Lymph node involvement, pathological stage	
N0	41 (56 %)
N1	18 (25 %)
N2	13 (18 %)
NX	1 (≈1 %)
Neoadjuvant therapy	
No	61 (84 %)
Yes	12 (16 %)
Adjuvant chemotherapy	
No	51 (70 %)
Yes	22 (30 %)
Time since last curative surgery and PET/CT scan	
<6 months	7 (10 %)
6–12 months	10 (14 %)
12–18 months	16 (22 %)
18–24 months	14 (19 %)
24–36 months	14 (19 %)
>36 months	12 (16 %)
Time since last postoperative imaging and PET/CT scan	
<3 months	22 (30 %)
3–6 months	14 (19 %)
6–12 months	15 (21 %)
>12 months	12 (16 %)
No postoperative imaging	10 (14 %)
Last postoperative imaging modality before PET/CT scan	
CT	55 (75 %)
MRI	4 (5 %)
PET/CT (not due to increasing CEA)	4 (5 %)
No postoperative imaging	10 (14 %)
CEA subgroups	
1 increase in CEA	30 (41 %)
2 consecutive increases in CEA	26 (36 %)
>2 consecutive increases in CEA	5 (7 %)
Persistently elevated CEA	12 (16 %)

*PET/CT* positron emission tomography/computed tomography, *MRI* magnetic resonance imaging, *CEA* carcinoembryonic antigen

### Imaging evaluation

The PET/CT was positive for CRC in 32 patients (44 %) and negative in 41 patients (56 %). The readers showed substantial agreement in their assessment with agreement in 89 % of the cases (Cohen’s kappa 0.778). A total of 41 malignant lesions were identified with a mean SUV_max_ of 8.6 (range 1.3–19.9). The distribution of FDG lesions assessed as positive for recurrent CRC is shown in Table 2. The term “other locations” included seven FDG-positive lesions located in distant lymph nodes (2 cases), abdominal wall (2), peritoneal carcinosis (2), and the top of the vagina. Most PET/CT positive lesions have SUV_max_ >5 (*n* = 33). In comparison to other regions, pulmonary lesions were frequently small with half of them with SUV_max_ <5. Two patients had malignant infiltrates in the lungs on CT below the detection limit of PET.

**Table 2:** Distribution of SUV_max_ assessed as positive for recurrent CRC on PET/CT

	Anatomical area					
	Local	Locoregional	Hepatic	Pulmonary	Other locations	All locations
SUV_max_ mean (range)	7.8 (2.9–18.8)	8.3 (6.2–10.0)	10.0 (3.3–19.9)	5.5 (1.3–13.3)	12.0 (8.1–19.5)	8.6 (1.3–19.9)
Lesions, number	8	7	10	9	7	41
SUV_max_ ≤2.5	0	0	0	2	0	2
SUV_max_ >2.5/<5.0	2	0	2	2	0	6
SUV_max_ ≥5.0	6	7	8	5	7	33

*SUV*_*max*_ maximum standardized uptake value

### Patient-based analysis

With regard to recurrent CRC, there were 30 true positive and 36 true negatives cases, two were false positive, and five were false negative cases, resulting in a PET/CT sensitivity of 85.7 % and specificity of 94.7 %. The positive predictive value (PPV) was 93.8 %, and the negative predictive value (NPV) was 87.8 %.

The two false positive cases were due to inflammation. Both patients were referred with a single rising CEA (3.1 and 5.2 μg/L).

Five false negative cases were observed by PET/CT of which four patients were positive for CRC by subsequent pathology performed 6–24 months after PET/CT (median 15 months). Two patients had one rising CEA (5.1 and 5.9 μg/L) and confirmed pulmonary metastases. Two patients had two consecutive increases in CEA (peak 4.4 and 5.9 μg/L) with liver metastases and pelvic recurrence, respectively. One patient had persistently elevated CEA (5.8 μg/L) and presented with liver metastases.

### Prior imaging

**Fig. 1:**
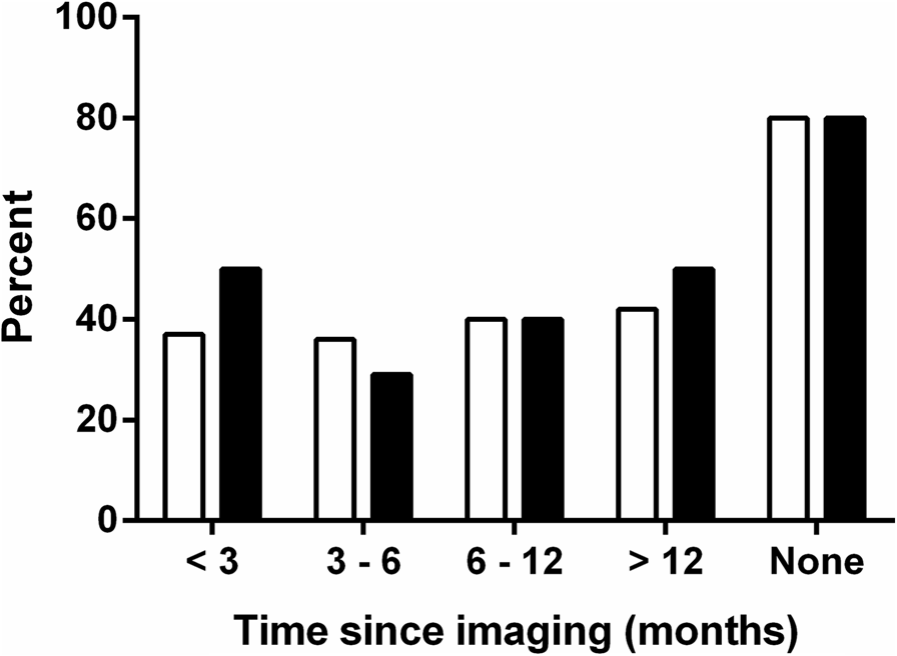
Patient-based PET/CT outcome and CRC recurrence in relation to time since the last postoperative imaging. The population was subdivided into five groups: <3 months since imaging (*n* = 22), 3–6 months since imaging (*n* = 14), 6–12 months since imaging (*n* = 15), >12 months since imaging (*n* = 12) and no postoperative imaging (*n* = 10). White boxes: PET/CT-positive, black boxes: Verified CRC recurrence

Almost one-third of the patients had post-operative imaging within three months from the actual PET/CT (Table 1) with a median time of 4 months (range <1–59 months). Most prior investigations were contrast-enhanced CT. The outcome of PET/CT versus the time since the last post-operative imaging is shown in Fig. 1. PET/CT was the first post-operative imaging modality after surgery in ten patients. There was no significant differences in PET/CT positivity (*p* = 0.18) or confirmed CRC (*p* = 0.16) with increasing time since last imaging; there was no significant differences in PET/CT sensitivity (73–100 %, *p* = 0.47) or specificity (89–100 %, *p* = 0.71) with time since last imaging.

### CEA

The diagnostic properties of PET/CT were analyzed in subgroups of patients with rising or elevated CEA (Table 3). The median CEA was 5.6 μg/L (range 1.5–164.0). There were a notable number of PET/CT positive patients and patients with confirmed CRC among patient with 1, 2, and >2 CEA increases as well as among patient with persistently elevated CEA. There was a significant difference among the proportion of patients with a positive PET/CT (*p* = 0.02) among these groups, but no clear statistical difference in confirmed CRC (*p* = 0.26).

**Table 3:** PET/CT outcome and CRC recurrence in CEA subgroups

	CEA subgroups			
	1 increase (*n* = 30)	2 increases (*n* = 26)	>2 increases (*n* = 5)	Persistently elevated (*n* = 12)
Number of PET/CT-positive patients	19 (63 %)	7 (27 %)	3 (60 %)	3 (25 %)
Number of patients with CRC recurrence	18 (60 %)	10 (38 %)	3 (60 %)	4 (33 %)
Median CEA, μg/L	6.75	4.85	4.0	5.7
CEA range, μg/L	1.7–164.0	2.3–16.4	1.5–6.1	3.4–7.8

*PET/CT* positron emission tomography/computed tomography, *CRC* colorectal cancer, *CEA* carcinoembryonic antigen

**Fig. 2:**
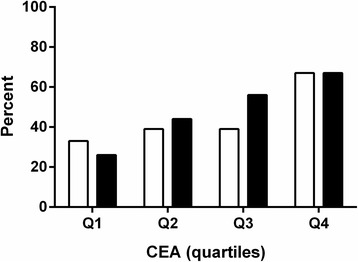
Patient-based PET/CT outcome and CRC recurrence in relation to CEA quartiles. The population was subdivided into four groups: Group Q1 (*n* = 19), below the first quartile; group Q2 (*n* = 18), between the first quartile and the median; group Q3 (*n* = 18), between the median and the third quartile; and group Q4 (*n* = 18), above the third quartile. White boxes: PET/CT-positive, black boxes: Verified CRC recurrence

The impact of the CEA level at referral is shown in Fig. 2. The patients were divided into quartiles. The median CEA levels in the four quartiles were: Q1 (4.0 μg/L, *n* = 19), Q2 (5.1 μg/L, *n* = 18), Q3 (6.1 μg/L, *n* = 18), and Q4 (13.1 μg/L, *n* = 18). The data confirmed a significant association of CEA level with a positive PET/CT outcome (*p* = 0.04) and recurrent CRC (*p* = 0.01).

**Fig. 3:**
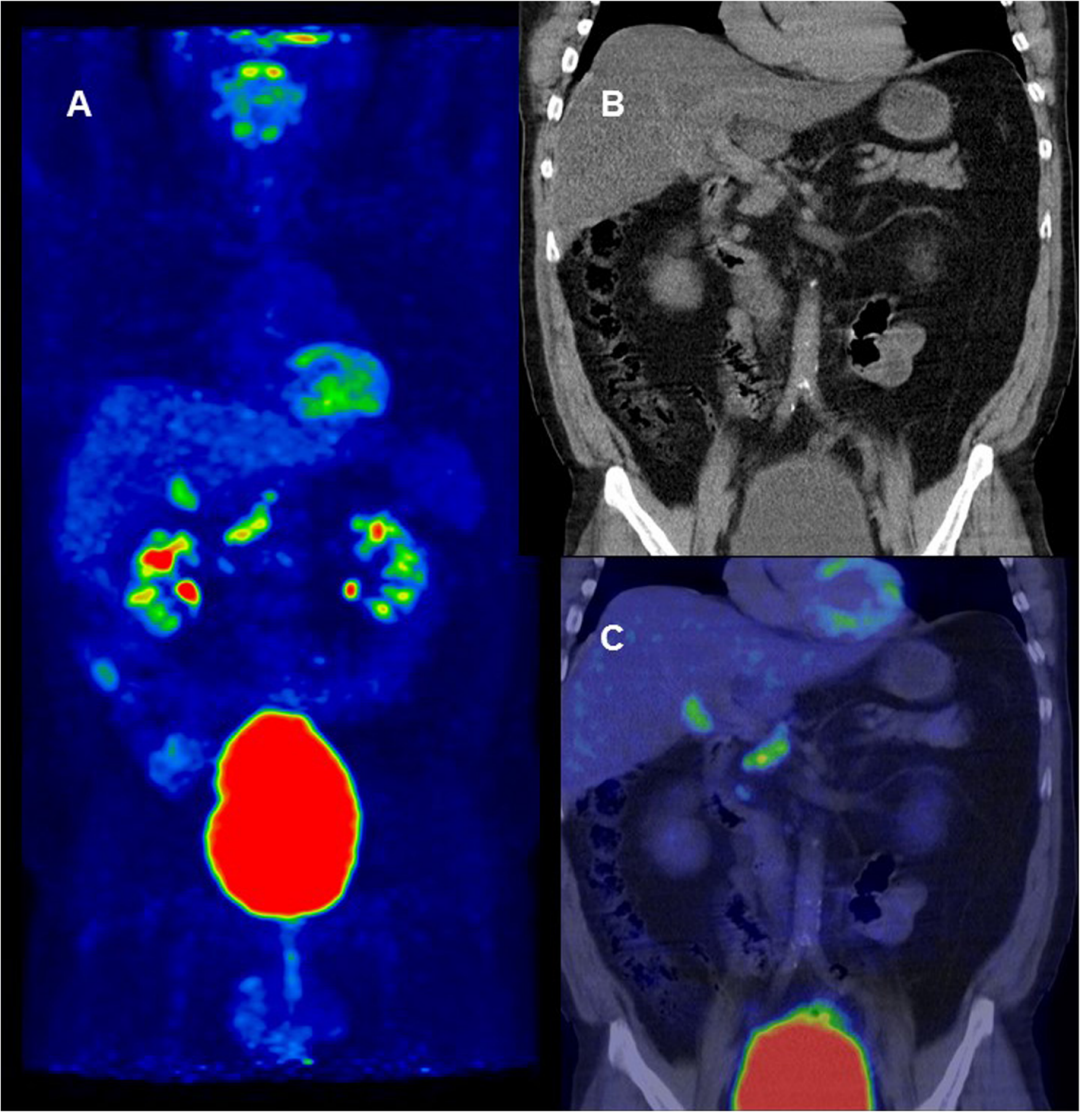
Illustrative example of PET/CT for the detection of recurrent CRC. A 54-year-old man with adenocarcinoma in the sigmoid colon (pT4, pN2, M0) received three cycles of neoadjuvant chemotherapy followed by radical surgery in May 2010 and five cycles of adjuvant chemotherapy. The patient presented with one significant increase in CEA from 1.4 μg/L (May 2011) to 5.1 μg/L (November 2011), and he underwent an ^18^F-FDG PET/CT scan with low-dose CT. Intraabdominal as well as retroperitoneal lymph nodes were found to exhibit pathological FDG uptake (SUV_max_ = 8.1), especially at foci near the liver hilus and portacaval area (PET anterior view (**a**); CT coronal image (**b**); PET/CT coronal fused image (**c**). The patient had recurrence on the basis of radiological and clinical follow-up and died shortly after the study period. A conventional CT scan of the thorax, abdomen and pelvis in usual surveillance was carried out 41 days earlier with no evidence of recurrence or metastasis

Diagnostic properties of ^18^F-FDG PET/CT for the detection of recurrent CRC were calculated in the divided CEA subgroups and quartiles (Table 4). There were no significant differences with regards to sensitivity or specificity among CEA types (p-values 0.60 and 0.38) or quartiles (p-values 0.26 and 0.21). PPV as well as NPV was excellent in patients with the highest CEA levels at referral (Q3 and Q4), but of clinical interest also in patients with lower CEA levels. PPV was excellent in all types of CEA increases but 1 single rise. Figure 3 shows an illustrative example of PET/CT for the detection of recurrent CRC.

**Table 4:** ^18^F-FDG PET/CT in the detection of CRC recurrence in patients with different CEA patterns

		PET/CT diagnostic properties			
		Sensitivity	Specificity	PPV	NPV
CEA quartiles	Q1 (*n* = 19)	100.0 %	92.9 %	83.3 %	100.0 %
	Q2 (*n* = 18)	75.0 %	90.0 %	85.7 %	81.8 %
	Q3 (*n* = 18)	70.0 %	100.0 %	100.0 %	72.7 %
	Q4 (*n* = 18)	100.0 %	100.0 %	100.0 %	100.0 %
CEA rise type	1 increase (*n* = 30)	94.4 %	83.3 %	89.5 %	90.9 %
	2 increases (*n* = 26)	70.0 %	100.0 %	100.0 %	84.2 %
	>2 increases (*n* = 5)	100.0 %	100.0 %	100.0 %	100.0 %
	Persistently elevated (*n* = 12)	75.0 %	100.0 %	100.0 %	88.9 %

^*18*^ *F-FDG PET/CT* ^18^F-fluorodeoxyglucose positron emission tomography/computed tomography, *CRC* colorectal cancer, *CEA* carcinoembryonic antigen, *PPV* positive predictive value, *NPV* negative predictive value

### Diagnosis of non-CRC malignancies

Two patients were PET/CT positive for primary lung cancer as confirmed by pathology. Furthermore, one patient was categorized as PET/CT positive for both recurrent CRC (liver metastasis) and primary lung cancer, with both lesions confirmed by pathology. Two of these patients were referred with one rising CEA (7.0 and 42.7 μg/L) and one patient had two consecutive increases in CEA (peak 16.4 μg/L). The diagnostic properties of ^18^F-FDG PET/CT for any malignancy (CRC and non-CRC) showed a sensitivity of 86.5 %, a specificity of 91.9 %, a PPV of 94.1 %, and an NPV of 87.2 %.

## Discussion

It has been our institutional practice to perform ^18^F-FDG PET/CT as the first imaging on suspicion of recurrent CRC due to unexplained rising CEA. If prior imaging was performed approximately more than one month ago, it was institutional practice to perform the PET/CT with contrast-enhancement. The results showed excellent sensitivity and specificity as well as predictive values for recurrent CRC in a largely asymptomatic population. This retrospective study included a rigorous selection of eligible patients from a 7-year consecutive population. Curative surgery was validated by records and pathology specimens. Strict and specific criteria for the presence or absence of recurrent CRC were applied. PET/CT, mostly performed with contrast-enhancement, were independently evaluated with substantial agreement by two readers blinded to any clinical, laboratory or pathology data accumulated after the date of PET/CT. Our study demonstrated excellent diagnostic accuracy of ^18^F-FDG PET/CT as the primary imaging tool in patients with rising CEA and suspicion of recurrent CRC.

The value of follow-up of CRC has been questioned for years, including the value of scheduled and clinical required visits [11]. A recent prospective, non-randomized study demonstrated that the majority of patients with recurrent CRC were identified at scheduled visits in a strict follow-up program. In that trial, CT and FDG-PET combined had better diagnostic properties than other imaging modalities [12]. A very recent randomized trial with more than 1,200 patients showed no benefit of combining serial CEA measurements with CT versus CT or CEA alone [13]. Our results indicated a high sensitivity and specificity of ^18^F-FDG PET/CT in detecting recurrent CRC, largely regardless of CEA levels and the level of CEA increases. These data are largely in line with findings from other retrospective studies [14–17]. We are only aware of one prior study of PET/CT as the first imaging on suspicion on recurrent CRC [18]. That study required CEA levels above 5.0 μg/L, provided limited information about the research methodology, but it showed diagnostic outcome similar to our data.

A notable proportion of patients with negative findings on routine CT performed within 3 to 6 months of referral presented with a positive PET/CT. Even though this study was not a comparison of CT vs. PET/CT, these findings are in line with several studies. A number of studies have compared ^18^FDG PET/CT and conventional CT in patients with increasing or elevated CEA and have shown a better overall detection rate for PET/CT [19] and higher sensitivity [20]. Our findings of recurrent malignancy which has not been observed at recent routine CT is interesting, but the data do not allow us to conclude that PET/CT is superior to conventional CT.

Recurrent CRC was found in approximately 50 percent of our study population, and PET was positive in the majority of the patients. However, we did not include patient without any rise of CEA. So it would not be appropriate to claim superiority of PET over CEA for the detection of recurrent CRC. The role of CEA as gatekeeper can be discussed. Makis et al., identified 99 patients with true positive PET for recurrent CRC of which only 65 patients presented with elevated CEA [21]. In contrast, in a small study with some methodological concerns, Amin et al., found only 1 in every 10 patients with unexplained rising CEA to have a true positive PET for CRC [22]. In summary, even though unexplained rising CEA is not a specific biomarker for recurrent CRC [6], CEA will likely still be considered a valuable biomarker and gatekeeper for PET.

The proportion of positive PET/CT findings and verified CRC recurrence increased with absolute CEA levels, which is consistent with prior studies [19, 23]. It could be debated if an absolute value of CEA should be required for referral for PET/CT [17, 21]. A recent meta-analysis showed upper limits of normal in the original papers ranged from 3 to 15 μg/L [24]. Tan et al., suggested a cut-off of 2.2 μg/L to demonstrate a high specificity (90 %) though limited sensitivity (64 %) for the diagnosis of recurrent CRC [24]. Most of our patients presented with absolute CEA levels above of the upper limit of 3 μg/L, the institutional upper limit of normal for non-smokers. Our data do not allow us to draw conclusions about the role of PET/CT in patients with normal levels of CEA. However, other reports have documented PET-positive, confirmed CRC recurrence in patients with normal CEA values [21].

In addition to the absolute CEA levels, the pattern of CEA rise is relevant to consider. The National Comprehensive Cancer Network considers serial CEA increases as an indication for workup for recurrence [25]. The advantages of serial versus single CEA increases have been reviewed by Goldstein et al. [6]. It was documented that serial measurements are more effective than clinical evaluation or other diagnostic modalities in postoperative detection of patients with metastatic disease, with the highest sensitivity for liver metastases. However, there are conflicting data about the diagnostic role of serial versus single CEA elevations [26, 27]. We identified a notable proportion of patients with recurrent CRC with 1, 2, >2 or persistently elevated CEA levels. Among our patients, the median CEA at referral was higher (and with a much broader range) in patients with one increase than in patients with two measurements. This may explain the low rate of positive PET/CT scans in the latter group. The time intervals between CEA measurements should also be considered. Patients with a single large increase in CEA may be referred directly for PET whereas a minor increase led to referral only when the increasing trend has been confirmed. Thus we believe that ^18^F-FDG PET/CT can be usefully used as first imaging based on unexplained rising CEA, provided that CEA measurement and interpretation is consistent with individual patient’s clinical situation.

We are aware of methodological issues with this study, mostly related to the retrospective nature of the study. This includes variability in the time since surgery, time and type of the latest diagnostic imaging before PET/CT, CEA measurements, and the risk of recurrence after surgery. However, we applied strict criteria for the definitions of curative surgery, PET/CT scans were blindly re-read with consistency among readers, and there was adequate follow up in all patients. Lastly, most patients with recurrent CRC were biopsy-proven. The 24 month clinical observation period was selected since it was observed that a number of patients presented with biopsy-proven CRC between 1 and 2 years after the PET/CT. We are aware of the potential bias of a long time interval from PET/CT to a final diagnosis. A disease classification based on histopathology or clinical follow-up 2 years after imaging may false classify some patients as false negative, although they could be true negative at the time of the PET/CT scan.

## Conclusions

We conclude that ^18^F-FDG PET/CT have high diagnostic value and can be used as the first choice in the detection of recurrent CRC in patients with unexplained rising CEA, even in patients with a recent normal routine CT.

## Abbreviations

^18^F-FDG: ^18^F-fluorodeoxyglucose
CEA: Carcinoembryonic antigen
CECT: Contrast-enhanced computed tomography
CRC: Colorectal cancer
CT: Computed tomography
NPV: Negative predictive value
PET/CT: Positron emission tomography/computed tomography
PPV: Positive predictive value
SUV_max_: Maximum standardized uptake value
